# Dropped head syndrome suspected due to oxaliplatin used in adjuvant chemotherapy for gastric cancer: a case report

**DOI:** 10.1186/s40792-024-01976-w

**Published:** 2024-07-25

**Authors:** Yuta Marunaka, Takuma Ohashi, Takeshi Kubota, Keiji Nishibeppu, Hirotaka Konishi, Atsushi Shiozaki, Hitoshi Fujiwara, Eigo Otsuji

**Affiliations:** Division of Digestive Surgery, Department of Surgery, Kyoto Prefectural University of Medicine, 465 Kajii-Cho, Kawaramachihirokoji, Kamigyo-Ku, Kyoto, Japan

**Keywords:** Adjuvant chemotherapy, Oxaliplatin, Dropped head syndrome

## Abstract

**Background:**

Dropped head syndrome (DHS) is caused by dysfunction of the cervical musculature. It is classified into two groups according to the cause: one is weakness of the neck extensors and the other is hypercontraction of the cervical flexors associated with Parkinson’s disease and other disorders. Although some drugs have previously been reported as suspected causes of DHS, we are unaware of any reports in which oxaliplatin was suspected. In this report, we describe a case of DHS during adjuvant chemotherapy for gastric cancer, along with a review of the relevant literature.

**Case presentation:**

A 72-year-old man was diagnosed with gastric cancer, cT3N0M0 cStage IIB, and underwent laparoscopic total gastrectomy with D2 lymphnode dissection and Roux-en-Y reconstruction. The operative time was 311 min, intraoperative blood loss was 40 g, and he was discharged without any post-operative complications. The histopathological diagnosis was pT4aN2M0 pStage IIIA, and S-1 + oxaliplatin (SOX) therapy was started as adjuvant chemotherapy. On the 4th course of SOX, he complained of neck heaviness and a blood test revealed that his creatine kinase (CK) level was elevated to 2464 IU/L. After consultation with an orthopedic surgeon and a neurologist, DHS due to localized cervical extensor myositis was suspected. Therefore, the 6th course of SOX was postponed, and 30 mg of oral steroids were initiated. His symptoms improved, and his CK level decreased within 2 weeks. After resuming S-1 monotherapy and tapering off oral steroids, no recurrence of symptoms has been observed.

**Conclusions:**

We experienced one case of DHS during adjuvant chemotherapy for gastric cancer. If DHS develops after starting oxaliplatin, involvement of the drug should be suspected, and discontinuation of chemotherapy and introduction of oral steroids should be considered.

## Background

Gastric cancer continues to have the fifth highest morbidity and mortality rates in the world [1]. However, treatment outcomes for gastric cancer have been improving due to recent medical advances, including chemotherapy [2]. The ARTIST2 trial demonstrated that SOX (S-1 + oxaliplatin) was superior to SP (S-1 + cisplatin) in terms of 3-year disease-free survival, when used as adjuvant chemotherapy in Stage II or III advanced gastric cancer [3]. Oxaliplatin is also widely used to treat other gastrointestinal cancers [4]. Adverse events with oxaliplatin have been reported, for example peripheral neuropathy [5], but dropped head syndrome (DHS) is rare.

DHS is caused by dysfunction of the cervical musculature. A patient’s neck continually drops and they are unable to look forward horizontally. It can be caused by Parkinson’s disease, autoimmune diseases, and drug-related adverse events [6]. Dipeptidyl peptidase-4 inhibitors and selumetinib have been reported to be suspected causes of DHS [7, 8]. In this report, we describe a case of DHS suspected to have been caused by oxaliplatin, and review the relevant literature.

## Case presentation

A 72-year-old male presented to his local doctor with abdominal discomfort. Upper gastrointestinal endoscopy revealed he had advanced gastric cancer. He was referred to our department (Division of Digestive Surgery, Department of Surgery, Kyoto Prefectural University of Medicine) for close examination and treatment. A physical examination was performed and the findings were as follows: height, 177.7 cm; body weight, 60.7 kg; body mass index (BMI), 19.2 kg/m^2^; blood pressure, 132/88 mmHg; pulse, 69 beats per minute; temperature, 36.8 °C; and his abdomen was flat and soft with no tenderness. Blood test results returned no significant findings in liver function, renal function, or electrolytes; tumor markers (CEA, CA19-9) were within the normal range. Upper gastrointestinal endoscopy showed swelling folds extending from the upper to the lower gastric body, and a biopsy revealed signet ring cell carcinoma. Gastrofluoroscopy showed poor extension and luminal narrowing from the upper to the lower gastric body. A contrast-enhanced computed tomography scan of the abdomen showed wall thickening with the contrast effect seen in the gastric body; there was no evidence of regional lymph node metastasis or distant metastasis.

Based on the above findings, he was diagnosed with gastric cancer UM, circ, Type 4, 60 mm, sig, cT3N0M0 cStage IIB [9]. He underwent laparoscopic total gastrectomy with D2 lymph node dissection and Roux-en Y reconstruction. The operative time was 311 min, and intraoperative blood loss was 40 g. Histopathological findings were por2 > sig, UML, Less, Type 4, 15 × 14.7 cm pT4a(SE), INFc, Ly1b, V0, pPM0, pDM0, pN2 (station 3, 7). The post-operative diagnosis was pT4aN2cM0 pStage IIIA [9].

**Fig. 1:**
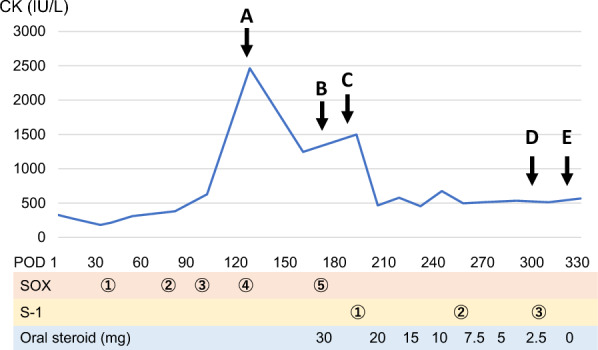
Post-operative course. A (POD124): the patient complained of neck heaviness and creatine kinase (CK) elevation was observed. B (POD170): he was diagnosed with dropped head syndrome (DHS). Future S-1 + oxaliplatin (SOX) therapy was discontinued, and oral steroids were started. C (POD184): his symptoms improved and CK decreased, so S-1 monotherapy was resumed. D (POD300): no recurrence of his gastric cancer was observed up until the end of his observation period. E (POD321): oral steroids were terminated without any symptom recurrence or CK re-elevation

**Fig. 2:**
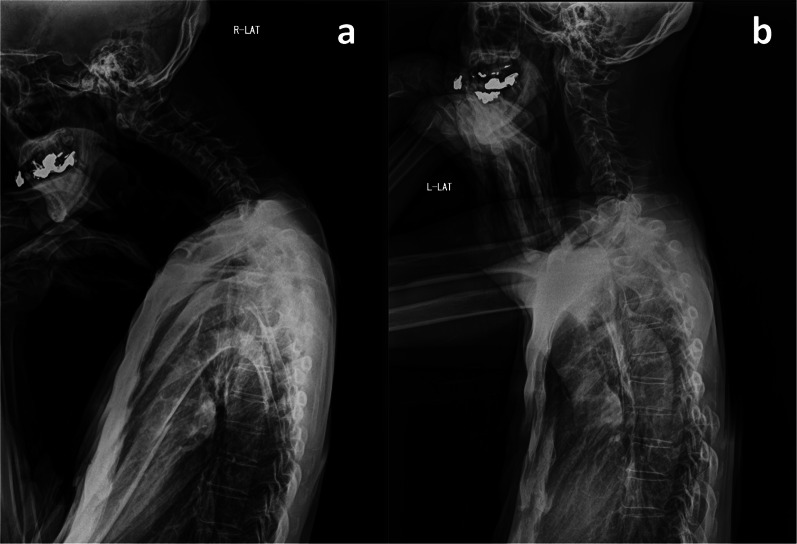
X-ray examination. Cervical–sagittal vertical axis (C-SVA) is defined as the distance between a perpendicular line through the center of the second cervical vertebra and the posterior superior margin of the seventh cervical vertebra. **a** (POD170): C-SVA was 68 mm; a C-SVA larger than 40 mm indicates an irregular cervical spine alignment. **b** (POD184): after S-1 + oxaliplatin (SOX) therapy was discontinued, C-SVA improved to 29 mm and his symptoms improved

**Fig. 3:**
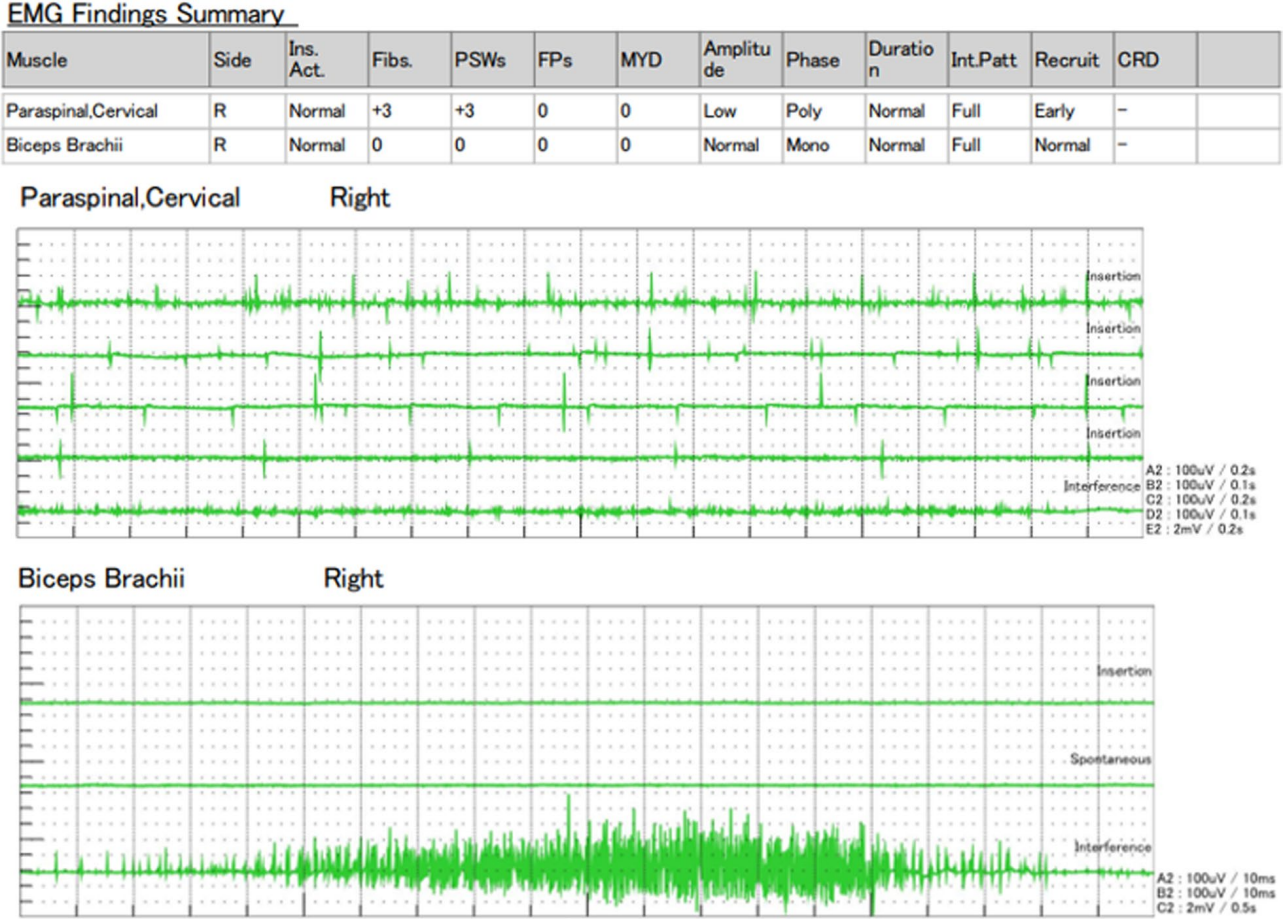
Electromyographic examination. In the cervical paraspinal muscle, the motor unit potential exhibited low amplitude polymorphism, and positive sharp wave and fibrillations were observed. This indicates the presence of active denervation, especially myositis. These findings were not observed in the biceps brachii muscle

Post-operative course is described in Fig. 1. He was discharged on post-operative day (POD) 8 without any complications. SOX therapy, as adjuvant chemotherapy, was started on POD 33. On POD 124 (the 4th course of SOX), the patient complained of neck heaviness. A blood test revealed that his creatine kinase (CK) level was elevated to 2464 IU/L, sodium was elevated to 146 mmol/L, and potassium was elevated to 6.0 mmol/L. SOX therapy was not initially suspected as the cause of his symptoms, so his 5th course was continued. However, his symptoms persisted and started to interfere with his daily life. Therefore, on POD 170, he was referred to an orthopedic surgeon and a neurologist. X-ray examination revealed irregularities in cervical spine alignment, and he was diagnosed with DHS (Fig. 2a). There was increased luminosity of the cervical extensor muscle on echographic examination; a finding of active denervation, especially myositis, confined to the cervical paraspinal muscle on electromyography (Fig. 3); and anti-SRP and anti-HMGCR antibody tests returned negative results. Therefore, dystonia and autoimmune myositis were ruled out, and drug-induced DHS due to SOX therapy was suspected. Consequently, his 6th course of SOX was postponed, and he was started on 30 mg oral steroids. On POD 184, his symptoms improved and his CK level decreased (Fig. 2b), so S-1 monotherapy was resumed. After three courses of S-1 monotherapy, no recurrence of his gastric cancer was observed up until the end of his observation period. Oral steroids were tapered off and terminated at POD 321, without any recurrence of symptoms or CK re-elevation.

## Discussion

DHS comprises a series of syndromes that involve severe dysfunction of the cervical extensor musculature; it was first described by Suarez and Kelly in 1992 [10]. Endo et al. reported that DHS is mainly observed in elderly women [11]. The causes of DHS can be broadly classified into two groups: those with weakness of the cervical extensor muscle group and those with strong contraction of the cervical flexor muscle. The former includes polymyositis, signal recognition particle (SRP)-positive myositis, myasthenia gravis, hypothyroidism, and localized cervical extensor myositis; the latter includes Parkinson’s disease and cervical dystonia [12]. Although treatment of the etiologic disease is a priority in cases of DHS, it is often difficult to identify the causative disease, and conservative treatment is often used, such as orthotics and medication [6]. Surgical treatment may be indicated if symptoms such as difficulty in opening the mouth and in swallowing are severe [6]. In our case, drug-induced localized extensor myositis of the cervical muscles was suspected because of the increased luminosity of the cervical extensor muscle on echographic examination; the finding of active denervation, especially myositis, confined to the cervical paraspinal muscle on electromyography; and the negative results of anti-SRP and anti-HMGCR antibody tests. After consultation with an orthopedic surgeon and a neurologist, the suspected drugs, S-1 and oxaliplatin, were discontinued, and the patient was started on oral steroids. After re-starting S-1 alone he experienced no relapse of symptoms, therefore we concluded that oxaliplatin was the cause of his symptoms.

Although myelosuppression and peripheral neuropathy have been reported to be adverse events linked with oxaliplatin, myopathy, including DHS, is rare. Maruoka et al. reported rhabdomyolysis in the proximal part of the limbs due to oxaliplatin [13]. They suggested the following four pathologies as the possible cause of rhabdomyolysis: (1) direct drug-induced injury, (2) electrolyte abnormalities such as hypokalemia, (3) injury secondary to impaired consciousness, and (4) allergic reaction, but the exact mechanism is unknown. The mechanism was similarly unknown in our case, and we could not find any reports of symptoms confined to the neck region.

On the other hand, there have been several reports of drug-induced DHS. A search of PubMed and the NPO Japan Medical Abstracts Society (keywords: “drug-induced” or “chemotherapy” and “dropped head syndrome”, published between 2000 and 2022, excluding conference proceedings) returned seven cases [7, 8, 14–16]. The suspected drug was amantadine in one case, a dipeptidyl peptidase-4 inhibitor in two cases, selumetinib in three cases, and a combination of imatinib and binimetinib in one case (Table 1). In all cases, the only treatment was discontinuation of the suspect drugs, and symptoms improved within 10 to 30 days, with no symptom recurrence during the observation period. Oral steroids were started in one case, but that alone did not improve the patient’s symptoms. There have been no reports of platinum-based anticancer drugs causing DHS, as happened in our case, therefore further accumulation of evidence regarding cases and investigations into the relationship between platinum-based anticancer drugs and DHS are warranted. Based on our case, if DHS develops following oxaliplatin administration, discontinuation of chemotherapy and introduction of oral steroids should be considered.

**Table 1:** Reported cases of drug-induced dropped head syndrome

Authors	Year	Age	Sex	Primary illness	Suspect drug	Treatment	Time to improvement	Recurrence
Kajikawa et al.	2007	74	Male	Vascular parkinsonism	Amantadine	Drug suspension	10 days	None
Chen et al.	2012	71	Female	Choroidal melanoma	Selumetinib	Drug suspension	1 month	N/A
Chen et al.	2012	76	Female	Choroidal melanoma	Selumetinib	Drug suspension oral steroids	Unknown (oral steroids were ineffective)	N/A
Chen et al.	2012	73	Male	Choroidal melanoma	Selumetinib	Drug suspension	2 weeks	N/A
Akaishi et al.	2013	87	Male	Diabetes	DPP-4 inhibitor	Change of drug (voglibose)	4 weeks	None
Ota et al.	2021	63	Male	Diabetes	DPP-4 inhibitor	Drug suspension	10 days	None
Chi et al.	2022	N/A	N/A	Gastrointestinal stromal tumor	Combination of imatinib and binimetinib	Drug suspension	N/A	N/A
Our case	–	72	Male	Gastric cancer	Oxaliplatin	Drug suspension oral steroids	2 weeks	None

DPP-4 inhibitor: dipeptidyl peptidase-4 inhibitor; N/A: not available

## Conclusion

We experienced one case of DHS during adjuvant chemotherapy for gastric cancer. If DHS develops after starting oxaliplatin, the drug’s involvement should be suspected, and discontinuation of the drug and initiation of steroid therapy should be considered.

## Data Availability

The datasets used and/or analyzed in the current study are available from the corresponding author on reasonable request.

## Abbreviations

CK: Creatine kinase
C-SVA: Cervical–sagittal vertical axis
DHS: Dropped head syndrome
POD: Post-operative day
SOX: S-1 + oxaliplatin
SP: S-1 + cisplatin
SRP: Signal recognition particle
